# Submucosal tunneling endoscopic resection in retroflexion for gastric gastrointestinal stromal tumor of the fundus

**DOI:** 10.1055/a-2271-5816

**Published:** 2024-04-03

**Authors:** Georgios Mavrogenis, Marinos Chatzis, Anna Spanomanoli, Loukas Kaklamanis, Fateh Bazerbachi

**Affiliations:** 1 168211 Unit of Hybrid Interventional Endoscopy, Department of Gastroenterology, Mediterraneo Hospital, Athens, Greece; 2 168211 Department of Anesthesiology, Mediterraneo Hospital, Athens, Greece; 3 168211 Department of Pathology, Mediterraneo Hospital, Athens, Greece; 4 10589 Interventional Endoscopy Program, St. Cloud Hospital, CentraCare Health System, St Cloud, United States

Endoscopic resection of gastrointestinal stromal tumors of the fundus is challenging due to the need for full-thickness resection and the difficulties of defect closure. Submucosal tunneling under direct view through the esophagus can be carried out for esophageal tumors and gastric tumors of the cardia near the gastroesophageal junction. However, for tumors located at the fundus this is not feasible. In this video ( Video 1 ), we present the resection strategy for a gastric gastrointestinal stromal tumor (GIST) located at the fundus ( Fig. 1 ), utilizing submucosal tunneling endoscopic resection (STER) in retroflexion combined with clip-and-band traction, together with clip-and-loop closure of the mucosal defect.

**Fig. 1:**
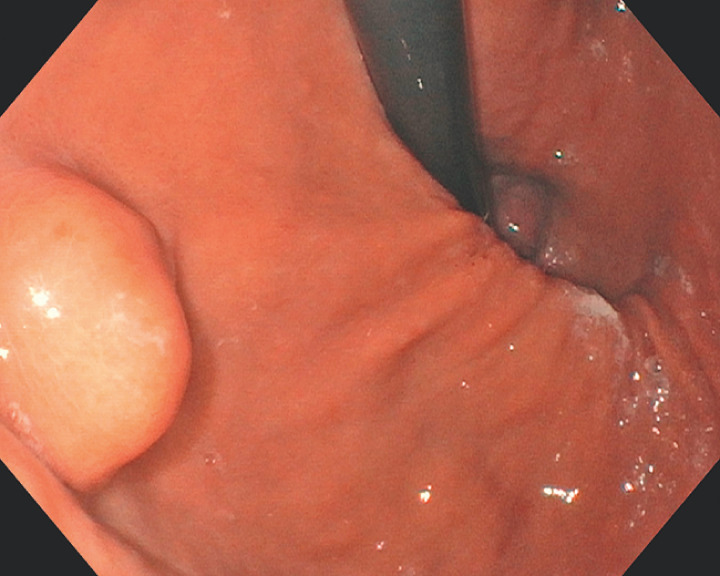
Submucosal tumor of the fundus distal to the cardia.

Submucosal tunneling endoscopic resection in retroflexion.Video 1

Initially, a horizontal incision was made 2 cm distal to the lesion in retroflexion with Flush Knife BTs 1.5 (Fujifilm, Tokyo, Japan). Then, a short pocket was created. In order to keep the mucosa away from the dissection field, a clip-and-band complex was applied ( Fig. 2 ) as previously described 1 . Cautious enucleation of the tumor was continued in retroflexion. Myotomy and detachment of the tumor from the muscle layer was performed using a Hook Knife (Olympus, Tokyo, Japan) ( Fig. 3 ). The presence of adipose tissue was visible at the end of the resection ( Fig. 4 ). After retrieval of the lesion, an endoloop was advanced over the scope and fixed in an open position with clips over the edges of the mucosal defect. Closure of the loop resulted in tight sealing of the mucosal defect ( Fig. 5 ). Next day a CT scan with oral contrast confirmed the absence of leakage. The patient was discharged 48h after the operation and followed a soft diet for 7 days. Histology showed a GIST with low mitotic index. At 1 year of follow-up the patient has no symptoms or signs of recurrence.

**Fig. 2:**
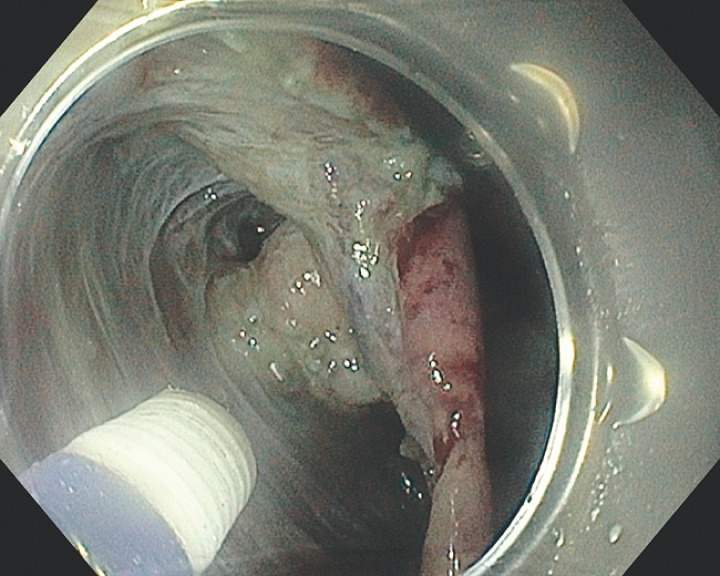
Clip-and-band countertraction is used to keep the mucosal flap away from the tumor.

**Fig. 3:**
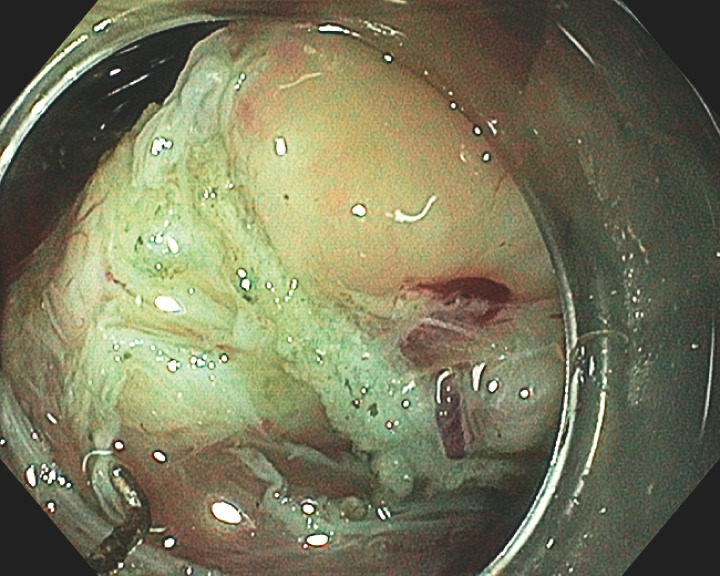
Detachment of the base of the tumor from the muscle layer using a Hook Knife.

**Fig. 4:**
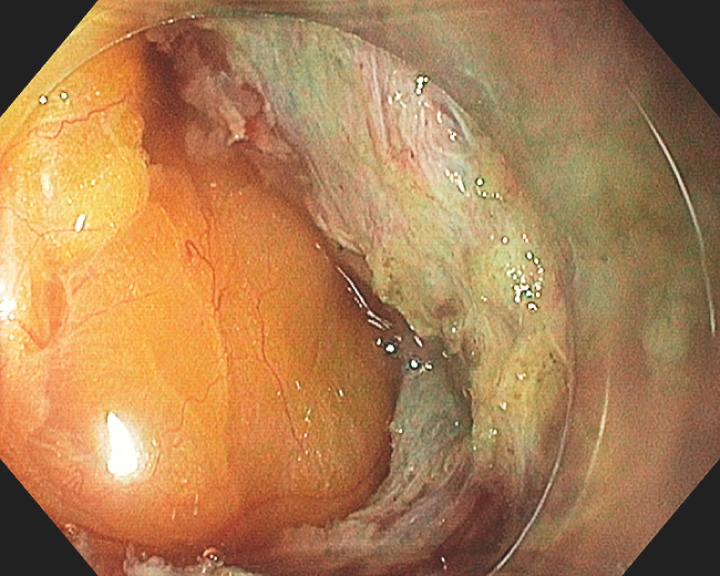
Wall defect at the end of the resection.

**Fig. 5:**
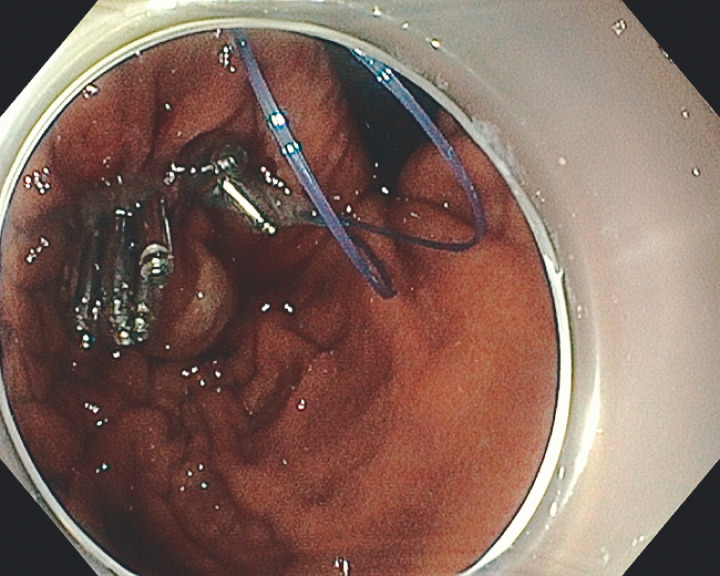
Tight closure of the mucosal defect using loop and clips.

In conclusion, we present a novel application of STER for challenging locations where straight tunneling is not feasible.

Endoscopy_UCTN_Code_TTT_1AO_2AG

## Notes

Endoscopy E-Videos https://eref.thieme.de/e-videos
*E-Videos* is an open access online section of the journal *Endoscopy* , reporting on interesting cases and new techniques in gastroenterological endoscopy. All papers include a high-quality video and are published with a Creative Commons CC-BY license. Endoscopy E-Videos qualify for HINARI discounts and waivers and eligibility is automatically checked during the submission process. We grant 100% waivers to articles whose corresponding authors are based in Group A countries and 50% waivers to those who are based in Group B countries as classified by Research4Life (see: https://www.research4life.org/access/eligibility/ ). This section has its own submission website at https://mc.manuscriptcentral.com/e-videos .
